# Efficacy and Safety of Low-Dose ATG Plus Basiliximab Induction in Deceased Donor Kidney Transplantation

**DOI:** 10.3389/ti.2025.15321

**Published:** 2025-10-13

**Authors:** Andrea Dello Strologo, Giulia Bartoli, Elisabetta Schifano, Maria Arena, Maria Paola Salerno, Patrizia Silvestri, Jacopo Romagnoli, Francesco Pesce, Giuseppe Grandaliano

**Affiliations:** ^1^ Azienda Sanitaria Locale Roma 6, Albano Laziale, Italy; ^2^ Department of Translational Medicine and Surgery, Università Cattolica del Sacro Cuore, Rome, Italy; ^3^ Nephrology and Dialysis Unit, Policlinico Universitario Tor Vergata, Rome, Italy; ^4^ Nephrology, Dialysis and Transplantation Unit, Fondazione Policlinico Universitario A.Gemelli IRCCS, Rome, Italy; ^5^ Renal Transplant Unit, Fondazione Policlinico Universitario A. Gemelli IRCCS, Rome, Italy; ^6^ Ospedale Fatebenefratelli Isola Tiberina - Gemelli Isola, Rome, Italy

**Keywords:** kidney transplant, immunosuppresion, new approaches, patient and graft survival, induction therapy

Dear Editors,

Kidney transplantation offers the best strategy for patients with End-Stage Kidney Disease (ESRD) [1]. The choice of induction therapy has always been a challenge for transplant clinicians. Strategies have been implemented to modulate the immune system, reduce rejection risk, and limit side effects such as infections and *de novo* tumors. Currently, most transplant centers use either a thymoglobulin (ATG)-based regimen (a depleting drug), which is the most immunosuppressive but has greater side effects, or a regimen based on anti-CD25 antibodies like Basiliximab which has fewer side effects but is less potent [2–4].

Over the years, our transplant center has sought an alternative solution. For this reason, we decided to implement a regimen involving the administration of both drugs but at reduced dosages. This strategy was hypothesized by Ruggenenti et al. [5] and has already been utilized and described by Hod et al. (though only in living-donor patients) [6] and by a US registry study [7]. The rationale was to exploit the benefits of both drugs, reducing tumors and infections without increasing acute rejection.

In this study, we evaluated the efficacy and safety of this approach compared to standard dose ATG alone and Basiliximab alone and the impact on biopsy proven 1-year rejections, occurrence of post-transplant neoplasia and infections, delayed graft function (DGF), graft and patient survival only in deceased donor patients.

We selected retrospectively 759 consecutive patients who received a single kidney transplant from a deceased donor at the Policlinico A. Gemelli Kidney Transplant Center, Rome, Italy, from 01/01/2001 to 31/12/2022. Patients were divided into three groups: 147 patients in the standard ATG group (7 mg/kg cumulative till day 7 post-transplantation (1 mg/kg/day)), 278 in the Basiliximab group (20 mg before surgery and another 20 mg 4 days post-surgery), and 334 in the low-dose ATG-Basiliximab group (ATG 1.5 mg/kg just before transplantation, 20 mg of Basiliximab mg pre-surgery and day 4). The choice of induction therapy was mainly based on the best clinical practices of the time. Specifically, ATG only was predominantly used from 2004 to 2010, Bas only from 2011 to 2016, and subsequently low-dose ATG and Bas. Baseline demographic, maintenance therapy and immunologic characteristics were comparable across the groups, although the ATG-Basiliximab group had a slightly lower HLA mean mismatch score (3.0 vs. 3.6 in ATG and 3.7 standard-Basiliximab). Drug levels and renal function were monitored according to institutional protocols [8].

Our findings yield various interesting results. First, biopsy-proven acute rejection (AR) occurred significantly more frequently in the Basiliximab group if compared to both ATG-containing groups. In fact, the low-dose ATG+Basiliximab group showed a significant protection (HR = 0.5031; 95%CI: 0.3276–0.7724; p = 0.0017). The ATG group showed a non-significant trend towards lower AR (HR = 0.5542; 95%CI: 0.3029–1.0140; p = 0.0555) (Figure 1A). This data supports previous data suggesting that a combination approach offers a synergistic immunosuppression [5]. On the other hand, this may be partially explained by the potential for excessive immunosuppression with high-dose ATG, which can lead to early dose reduction due to adverse effects. Such interruptions could blunt the protective effect of induction on early alloimmune activation. In contrast, the combined low-dose protocol may provide a more favorable balance between tolerability and immunologic efficacy [9].

**FIGURE 1:**
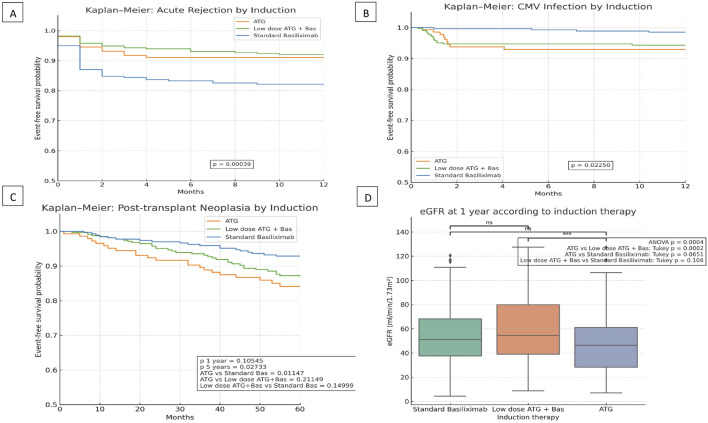
Clinical outcomes according to induction therapy in kidney transplant recipients: **(A)** Kaplan–Meier curve for biopsy-proven acute rejection within 12 months stratified by induction protocol. **(B)** Kaplan–Meier curve for CMV infection-free survival within 12 months by induction protocol. **(C)** Kaplan-Meier curve for post-transplant neoplasia-free survival by induction therapy. **(D)** Serum eGFR levels at 12 months post-transplantation across induction protocols.

Regarding the incidence of DGF alone, we noted that although the use of ATG and low-dose ATG was associated with a lower probability of developing DGF, this finding was not statistically significant.

In terms of graft function, patients in the low-dose ATG+Basiliximab group exhibited significantly better renal function at 1 year, as consistently indicated by higher eGFR levels (p = 0.0004) (Figure 1D). This superior graft function observed in the combined regimen group is highly likely linked to the lower incidence of acute rejection that characterizes this induction strategy. In a predefined sub-analysis stratifying recipients by age (<65 vs. ≥65 years), we observed that older patients consistently exhibited lower eGFR at 1 year irrespective of the induction regimen.

As anticipated and consistent with the known risks associated with T-cell-depleting agents, CMV infection was significantly more prevalent in both ATG-based regimens. Conversely, the Basiliximab-alone protocol was independently associated with a reduced CMV infection risk compared to the standard ATG protocol (HR 0.2256; 95% CI 0.0693–0.7348; p = 0.0135) (Figure 1B). This finding underscores the critical importance of implementing robust CMV prophylaxis and diligent monitoring strategies, especially when T-cell-depleting agents are employed in the immunosuppressive regimen [3, 9, 10].

From a safety perspective, we found no statistically significant differences in overall graft or patient survival among the three induction groups, although older recipient age emerged as a significant predictor of increased cancer risk and patient survival. As for the incidence of post-transplant malignancy only at 5 years, patients who received ATG only had a higher incidence of malignancies (Figure 1C).

The novelty and strength of this study is that it considers only patients with deceased donors, who are considered at higher risk of rejection and complications. In addition to this, we have well-matched the three groups, unlike the US registry study where this strategy was associated with worse outcomes, but it was often administered to patients with crucial differences in selection criteria and the specific dosing strategies employed was not indicated.

While acknowledging the inherent limitations of our study, including its retrospective, single-center design and the extended two-decade observational period, potentially introducing variability due to evolving standards of care, its strengths are considerable. These include the large cohort of only deceased donor recipients, and the detailed analysis of clinically relevant outcomes.

In conclusion, our extensive experience suggests that the use of a combined low-dose ATG and Basiliximab induction regimen offers a favorable balance between efficacy and safety in kidney transplant recipients from deceased donors. This specific protocol was consistently associated with improved one-year renal function and a tendency towards fewer acute rejections while maintaining manageable infectious risks compared with Basiliximab- or ATG-only strategies. Further prospective studies and well-designed randomized controlled trials are certainly warranted to validate these compelling findings and to further refine induction strategies based on individualized immunologic profiles, ultimately aiming to optimize clinical practices and enhance long-term patient outcomes in kidney transplantation.

## Data Availability

The raw data supporting the conclusions of this article will be made available by the authors, without undue reservation.
